# Pioglitazone Represents an Effective Therapeutic Target in Preventing Oxidative/Inflammatory Cochlear Damage Induced by Noise Exposure

**DOI:** 10.3389/fphar.2018.01103

**Published:** 2018-10-08

**Authors:** Fabiola Paciello, Anna Rita Fetoni, Rolando Rolesi, Matthew B. Wright, Claudio Grassi, Diana Troiani, Gaetano Paludetti

**Affiliations:** ^1^Fondazione Policlinico Universitario A. Gemelli, IRCCS, Rome, Italy; ^2^Institute of Otolaryngology, Università Cattolica del Sacro Cuore, Rome, Italy; ^3^Institute of Cell Biology and Neurobiology, CNR, Rome, Italy; ^4^Strekin AG, Basel, Switzerland; ^5^Institute of Human Physiology, Università Cattolica del Sacro Cuore, Rome, Italy

**Keywords:** PPAR agonist, acoustic trauma, antioxidant, antinflammatory, audiology, personalized medicine

## Abstract

Recent progress in hearing loss research has provided strong evidence for the imbalance of cellular redox status and inflammation as common predominant mechanisms of damage affecting the organ of Corti including noise induced hearing loss. The discovery of a protective molecule acting on both mechanisms is challenging. The thiazolidinediones, a class of antidiabetic drugs including pioglitazone and rosiglitazone, have demonstrated diverse pleiotrophic effects in many tissues where they exhibit anti-inflammatory, anti-proliferative, tissue protective effects and regulators of redox balance acting as agonist of peroxisome proliferator-activated receptors (PPARs). They are members of the family of ligand regulated nuclear hormone receptors that are also expressed in several cochlear cell types, including the outer hair cells. In this study, we investigated the protective capacity of pioglitazone in a model of noise-induced hearing loss in Wistar rats and the molecular mechanisms underlying this protective effects. Specifically, we employed a formulation of pioglitazone in a biocompatible thermogel providing rapid, uniform and sustained inner ear drug delivery via transtympanic injection. Following noise exposure (120 dB, 10 kHz, 1 h), different time schedules of treatment were employed: we explored the efficacy of pioglitazone given immediately (1 h) or at delayed time points (24 and 48 h) after noise exposure and the time course and extent of hearing recovery were assessed. We found that pioglitazone was able to protect auditory function at the mid-high frequencies and to limit cell death in the cochlear basal/middle turn, damaged by noise exposure. Immunofluorescence and western blot analysis provided evidence that pioglitazone mediates both anti-inflammatory and anti-oxidant effects by decreasing NF-κB and IL-1β expression in the cochlea and opposing the oxidative damage induced by noise insult. These results suggest that intratympanic pioglitazone can be considered a valid therapeutic strategy for attenuating noise-induced hearing loss and cochlear damage, reducing inflammatory signaling and restoring cochlear redox balance.

## Introduction

Recent progress in hearing loss research has provided strong evidence for common predominant mechanisms of damage affecting the organ of Corti: the imbalance of cellular redox status and inflammation. Although the detailed mechanisms and pathways activated by specific insults (e.g., noise, aging, aminoglycoside antibiotics, and anticancer drugs) have not been completely clarified, it has been demonstrated that overproduction of reactive oxygen species (ROS), with subsequent lipid peroxidation and cell damage and pro-inflammatory cytokine expression are central themes underlying hearing loss (Priuska and Schacht, 1995; Sergi et al., 2006). The ever-increasing understanding of cochlear biological systems is indeed providing a range of exciting novel biological targets, whose modulation may enable novel therapeutic options. We have previously demonstrated in animal models that, by targeting endogenous defense pathways, it is possible to attenuate hearing loss and cochlear damage. We have addressed redox unbalance in noise-induced hearing loss (NIHL) and drug-related ototoxicity and we provided evidence on cochlear protection by the activation of the Nrf-2/HO-1 axis through the supplementation of different antioxidant compounds such as coenzyme Q and the polyphenols ferulic acid, rosmarinic acid, and curcumin (Fetoni et al., 2010, 2014a, 2015). However, several issues such as those related to the absorption and distribution of polyphenols and other herbal products and interactions, with the lack of robust clinical trials demonstrating the efficacy and safety of these new formulations, limit their prescription as adjuvant or preventive therapy in conditions linked to oxidative stress (Mancuso, 2015). Therefore, preclinical studies are desirable focusing, not only on cytoprotective mechanisms, but also on upstream cytoxicity pathways in order to identify pathways and drugs able to address both oxidative stress and inflammation. Notably, in the last decades, a growing body of research has been dedicated to the PPARs, including PPARα, PPARβ/δ, and PPARγ. These receptors comprise a subfamily of the nuclear receptor superfamily of ligand-activated transcriptional factors that play critical physiological roles as lipid sensors and regulators of diverse metabolic pathways (Issemann and Green, 1990; Green, 1995; Kersten et al., 2000; Berger and Moller, 2002; Wang et al., 2013). Moreover, PPARs participate in the regulation of redox balance by upregulating the transcription of antioxidant related genes and by inhibiting the generation of ROS (Chen et al., 2012; Wang and Liu, 2012; Abushouk et al., 2017). To date, most studies have evaluated the role of PPARγ in major metabolic organs such as liver, adipocytes, pancreas, or skeletal muscles (Tontonoz and Spiegelman, 2008; Ahmadian et al., 2013), leading to target PPARγ for the treatment of type 2 diabetes with the development of the thiazolidinedione (TZD) class of drugs (Lehmann et al., 1995). The TZD drug pioglitazone, a PPARγ agonist approved by the FDA, is also known to decrease inflammatory mediators in patients with type 2 diabetes and coronary artery disease and to have favorable effects on vascular function (Wang et al., 2005; Forst et al., 2008; Cabrera et al., 2012; Kauppinen et al., 2013). Moreover, pioglitazone is the only approved PPARγ agonist with significant ability to cross the blood-brain barrier and has demonstrated neuroprotective benefits in models of Alzheimer’s disease, Parkinson’s disease, epilepsy and stroke (Kiaei et al., 2005; Hirsch and Hunot, 2009; Barnum and Tansey, 2010; Carta et al., 2011; Esposito and Cuzzocrea, 2011; Sekulic-Jablanovic et al., 2017).

Interestingly, the expression of PPARs in the cochlea have been reported recently (Sekulic-Jablanovic et al., 2017). PPARγ and PPARα were found to be highly expressed in several cochlear cell types, including inner and outer hair cells (HCs). Furthermore, in organ of Corti explants, pioglitazone and other related drugs effectively prevent gentamicin-induced toxicity to hair cells by blocking increased ROS, lipid peroxidation, the activation of pro-apoptotic caspases induced by gentamicin and by potentiating intrinsic cellular antioxidant defenses (Sekulic-Jablanovic et al., 2017). Moreover, PPAR agonists, such as pioglitazone, have also well-characterized pleiotropic effects, which may offer additional mechanisms of protection in hearing loss (Rizos et al., 2008).

Based on these findings, we aimed in the present work to evaluate the therapeutic potential of pioglitazone in the protection from NIHL. In order to ensure rapid and controlled drug delivery to the inner ear, we employed a formulation of pioglitazone in a biocompatible thermogel administered by transtympanic injection. Following noise exposure, immediate and delayed treatment regimens were employed and the time course and extent of hearing recovery were assessed. These results, along with effects on markers of cochlear oxidative stress and inflammation, suggest that pioglitazone is a compelling therapeutic target for the treatment of NIHL via multiple protective mechanisms, including early reduction in cochlear oxidative stress and long-term reduction in inflammatory signaling.

## Materials and Methods

### Animals

Male adult Wistar rats (UCSC Laboratories), 250–350 g, age 2 months, with intact Preyer’s reflex, were used for these studies. The experiments were performed on a total of 126 animals, randomized and assigned to 6 experimental groups as follows: (1) control animals (Ctrl group, *n* = 24); (2) animals exposed to acoustic trauma (Noise group, *n* = 24); (3) control animals treated with pioglitazone 1.2% suspension in the left ear and with matching vehicle in the right ear (Ctrl-Pio and Ctrl-vehicle group *n* = 6); (4) animals exposed to acoustic trauma and treated with pioglitazone 1.2% suspension in the left ear (Noise-Pio 1 h post group) and with vehicle in the right ear (Noise-vehicle 1 h post group) 1 h after acoustic trauma (*n* = 24); (5) animals exposed to acoustic trauma and treated with pioglitazone 1.2% suspension in the left ear (Noise-Pio 24 h post group) and with vehicle in the right ear (Noise-vehicle 24 h post group) 24 h after acoustic trauma (*n* = 24) and (6) animals exposed to acoustic trauma and treated with pioglitazone 1.2% suspension in the left ear (Noise-Pio 48 h post group) and with vehicle in the right ear (Noise-vehicle 48 h post group) 48 h after acoustic trauma (*n* = 24). Animals were housed two per cage at a controlled temperature (22–23°C) and constant humidity (60–75%), under a 12 h light/dark cycle, with food (Mucedola 4RF21, Italy) and water *ad libitum*, for the complete experimental period.

All efforts were made to minimize animal suffering and to reduce the number of animals, in accordance with the European Community Council Directive of 24 November 1986 (86/609/EEC). All procedures were performed in compliance with the Laboratory of Animal Care and Use Committee of the Catholic University, School of Medicine of Rome and were approved by the Italian Department of Health (*Ministero della Salute*).

### Acoustic Trauma

As described previously (Fetoni et al., 2015), acoustic trauma was induced by a continuous pure tone of 10 kHz generated by a waveform generator (LAG-120B, Leader, NY, United States) and amplified by an audio amplifier (A-307R, Pioneer, CA, United States). Under anesthesia (ketamine, 35 mg/kg and medetomidine-domitor, 0.25 mg/kg), all animals were placed in a soundproof room in a fixation cradle with their head gently maintained in a fixed position by a neck and nose ring. Then, they were exposed for 60 min to a 120 dB SPL sound presented to the ears in free field via loudspeakers (TW034X0, Audax, France) positioned at a distance of 10 cm in front of the animal’s head. Sound level was measured using a calibrated 1/4 in. microphone (Model 7017, ACO Pacific Inc., Belmont, CA, United States) and a calibrated preamplifier (Acoustic Interface System, ACO Pacific Inc.).

### Drug Administration

Animals received 1.2% pioglitazone suspension (Strekin AG, Basel, Switzerland) formulated in a temperature-sensitive gel formulation or the matching vehicle control gel (vehicle) via a single transtympanic injection. The vehicle formulation (Strekin AG, Basel, Switzerland) was composed of water, a detergent/surfactant, a buffer system to maintain the pH and a copolymer as the thermo-reversible component. The formulation was designed in accordance with published guidance to ensure it remains liquid at temperatures below 25°C and transitions to a viscous gel at temperatures above 35°C (Malmsten and Lindman, 1992). The final drug product was sterile. In order to evaluate the ability of pioglitazone to rescue hearing from acute acoustic trauma, animals were treated 1, 24, or 48 h after noise exposure. All surgical procedures were conducted under aseptic conditions, using aseptic techniques and under deep anesthesia (ketamine, 70 mg/kg and medetomidine-domitor, 0.5 mg/kg). In preparation for injection, the round window (RW) and the niche of RW were localized using a surgical microscope. Injection was performed using a 22 gauge, 1.5 inch, short beveled needle attached to a 100 μl luer-lock Hamilton syringe with the help of an operating microscope. A small perforation of the tympanic membrane was performed anterior to the malleus to equalize middle ear pressure (Fetoni et al., 2014b). A second perforation was made in a postero-inferior membrane region, through which 30 μl of either the pioglitazone 1.2% suspension or vehicle were injected at a constant rate (2 μl/s) into the RW niche area. Following dose administration, the animals were maintained for at least 10 min with the dosed ear facing upward in order to ensure test article delivery and contact with the RW. The animals were then turned onto the contralateral side and the opposite ear was then injected using the same procedure.

### Electrophysiological Measurements of Auditory Function

Hearing function was evaluated in all animals by measuring auditory brainstem responses (ABRs) at low (6 kHz), mid (12, 16, 20 kHz) and high (24, 32 kHz) frequencies. ABRs were assessed bilaterally prior to noise exposure to assure normal hearing and reassessed 1, 3, 7, 14 and 21 days after transtympanic drug injection to follow the course of recovery. During the procedure, animals were mildly anesthetized (ketamine, 35 mg/kg and medetomidine-domitor, 0.25 mg/kg) and placed in the anechoic room. As previously described (Fetoni et al., 2016), three electrodes were subcutaneously inserted into the right mastoid (active), vertex (reference) and left mastoid (ground). A PC-controlled TDT System 3 (Tucker– Davis Technologies, Alachua, FL, United States) data acquisition system with real time digital signal processing was used for auditory stimulus generation and ABR recording. Tone bursts, consisting of pure tones from 6 to 32 kHz (1 ms rise/fall time, 10 ms total duration, 20/s repetition rate), were presented monaurally in open field. Responses were filtered (0.3–3 kHz), digitized and averaged across 500 discrete samples at each frequency-sound level combination. Threshold values were defined as the lowest stimulus level (dB) that yielded a repeatable waveform-based onset.

### Hair Cell Count

Rhodamine–Phalloidin is a high-affinity F-actin probe conjugated to the red–orange fluorescent dye tetramethylrhodamine (TRITC). This stain is used to visualize the stereociliary arrays and cuticular plate of hair cells (HCs). After functional analyses (day 21 from drug injection) animals (*n* = 6/group) were sacrificed with a lethal dose of anesthetic, then cochleae were quickly removed and surface preparations of the organ of Corti were made for hair cell counting. Briefly, isolated cochleae were fixed with 10% buffered formalin for 4 h. Following removal of the bony capsule and the lateral wall tissues, the epithelium of the organ of Corti was separated from the bony modiolus and dissected into half-turns in 0.1 M PBS under a dissecting microscope. The preparations were then incubated with a solution containing 0.5% Triton X-100 and rhodamine-conjugated phalloidin (1:100 dilution; Molecular Probes, Invitrogen, Carlsbad, CA, United States) for 1 h at room temperature in the dark. The specimens were washed twice in PBS and then mounted on slides containing an antifade medium (ProLong, Cat. No. P36930, Invitrogen). Quantification of hair cells was performed with a confocal laser scanning system (Nikon Ti-E, Confocal Head A1 MP, Tokyo, Japan) using filters with an excitation of 516 nm and an emission of 543 nm. Z-stack series of 3–5 μm thickness were acquired as images of 1024 × 1024 pixels recorded at intervals of 0.5 μm. Images were taken at 40× magnification (Plan Fluo objective, Nikon). Hair cells were counted in segments of ∼250 μm sections along the length of the basilar membrane. The criterion to assess HC loss was the presence of either a dark spot and/or the typical phalangeal scar of supporting cells in the spaces previously filled by hair cells (Fetoni et al., 2013).

### Superoxide and 8-Isoprostane Detection

Dihydroethidium (DHE) and 8-Isoprostane immunostainings were used to assess superoxide anion and lipid peroxidation production, respectively. At day 7 after drug injection, a cohort of animals (6 animals/group) was sacrificed following ABR recording and the cochleae were quickly removed and fixed in 4% paraformaldehyde in PBS at 4°C and pH 7.5. The cochleae were then decalcified for 15 d in EDTA (10% EDTA, changed daily), incubated for 48 h in sucrose (30%), embedded in OCT (Killik, Bio-optica, Milan, Italy) and cryosectioned at a thickness of 12 μm (Cryostat CM 1950; SLEE medical GmbH, Mainz, Germany). For all immunofluorescence analysis, control experiments were performed by omitting the primary antibody during processing of tissues randomly selected across experimental groups. Staining was absent in cochlear samples, indicating neither spontaneous fluorescence nor non-specificity of antibody (data not shown). Tissues from all groups were always processed together during the procedures to limit variability related to antibody penetration, incubation time, post-sectioning age and condition of tissue.

#### Detection of Superoxide (DHE Assay)

DHE is a lipophilic cell-permeable dye that is rapidly oxidized to ethidium in the presence of free radical superoxide. The resulting ethidium intercalates into nDNA yielding a fluorescent signal indicating free radical level in the tissue (Fetoni et al., 2013). The cochlear specimens were incubated with 1 μM DHE (Cat. No. D23107, Invitrogen, Carlsbad, CA, United States) in PBS for 30 min at 37°C and then cover-slipped with an antifade medium (ProLong Gold, Invitrogen). Tissue fluorescence was imaged by two-photon excitation (792 nm, <140 fs, 90 MHz) performed by ultrafast tunable mode locked titanium: sapphire laser (Chameleon, Coherent). Images were captured at 20× magnification (Plan Apo objective, Nikon).

#### 8-Isoprostane Immunostaining

8-Isoprostane, a biomarker of oxidative stress, is a prostaglandin ((PG)-F2-like compound) that is produced *in vivo* by the free radical-catalyzed peroxidation of arachidonic acid. To detect 8-Isoprostane, slides were incubated in a blocking solution containing 1% fatty acid-free bovine serum albumin (BSA), 0.5% Triton X-100, and 10% normal goat serum in PBS for 1h at room temperature (Sigma-Aldrich, San Louis, MO, United States). The specimens were then incubated overnight at 4°C with rabbit polyclonal anti-8-Isoprostane primary antibody (Cat. No. IS20, Oxford Biomedical Research, Oxford, United Kingdom) diluted 1:100 in PBS. Slides were then washed twice in PBS and incubated at room temperature for 2 h, light-protected, in Alexa Fluor-labeled anti-rabbit secondary antibody (Alexa Fluor 488, IgG; Cat. No. A32731, Invitrogen) diluted 1:400 in 0.1 M PBS. Slides were washed once more in PBS, then counterstained with DAPI (blue fluorescence, 1:500, Cat. No. D1306, Invitrogen), to identify cell nuclei, for 20 min in the dark at room temperature. Slides were then cover-slipped with an antifade medium (ProLong Gold, Invitrogen). Images of 8-Isoprostane immunolabeled specimens (20×) were captured using a confocal laser-scanning microscope (Nikon Ti-E, Confocal Head A1 MP) equipped with argon/argon–krypton and helium/neon lasers for 488 and 519 excitation. DAPI staining was imaged by two-photon excitation (740 nm, <140 fs, 90 MHz) performed by an ultrafast tunable mode-locked titanium:sapphire laser. The 8-Isoprostane positive cells were identified by green fluorescence.

To perform semi-quantitative analysis of fluorescence signals, the fluorescence intensity of each area of interest, corresponding to spiral ganglion neurons (SGNs), organ of Corti and *stria vascularis*, was quantified with ImageJ (version 1.51s) on *N* = 12 slices selected randomly from 6 animals for each experimental group.

### NF-κB and IL-1β Immuno-Detection

Inflammatory cells in the cochlea are activated in response to various types of insults that induce cochlear damage. NF-κB is a critical regulator of the inducible expression of genes involved in immunity and inflammation, as well as in cell growth and death. We evaluated the effects of pioglitazone on NF-κB and IL-1β, a pro-inflammatory cytokine implicated in cochlear damage (Komeda et al., 1999; Satoh et al., 2002; Wang et al., 2003). Cochlear tissue (from 6 animals/group, day 7), prepared as described in the previous section, were incubated in a blocking solution containing 1% BSA, 0.5% Triton X-100 and 10% normal goat serum in PBS for 1h at room temperature. Slides were then incubated overnight at 4°C with a solution containing a rabbit monoclonal anti-NF-κB primary antibody (1:100, Cat. No. #8242, Cell Signaling, Denver, MA, United States) or a rabbit polyclonal anti-IL-1β primary antibody (1:100, Santa Cruz Tech., Cat. No. sc-7884, Santa Cruz, CA, United States) diluted 1:100 in PBS. Slides were washed twice in PBS and then incubated at room temperature for 2 h, light protected, in Alexa Fluor-labeled anti-rabbit secondary antibody (Alexa Fluor 546, Cat. No. A110040 and Alexa Fluor 488, IgG, Cat. No. A32731, Invitrogen) diluted 1:400 in 0.1 M PBS. After another wash in PBS, samples were counterstained with DAPI (blue fluorescence, 1:500) for 20 min in the dark at room temperature. Control experiments were performed by omitting the primary antibody during processing of tissue randomly selected across experimental groups, indicating neither spontaneous fluorescence nor non-specificity of antibody (data not shown). The slides were cover-slipped with an antifade medium (ProLong Gold, Invitrogen) and then images of NF-κB (red fluorescence) and IL-1β (green fluorescence) were captured using a confocal laser scanning microscope (Nikon Ti-E, Confocal Head A1 MP) equipped with argon/argon–krypton and helium/neon lasers for 488 and 519 excitation. Images were taken at 20× magnification. The analysis of fluorescence intensity of each area of interest, corresponding to SGNs, organ of Corti and *stria vascularis*, was quantified as indicated above.

### Western Blotting

Western blots were performed in order to confirm immunofluorescence results on NF-κB and IL-1β expression. Animals (6 animals/group) were sacrificed after the last ABR recording at day 7 after drug treatment. Similar to a method described previously (Fetoni et al., 2015), dissected cochleae were collected on ice, stored at -80°C, and then homogenized with RIPA buffer (Sigma-Aldrich). After centrifugation (12,000 rpm, 15 min, 4°C), protein concentrations in supernatants were determined using the Micro BCA kit (Pierce, Rockford, IL, United States). An aliquot of each supernatant containing 60 μg protein was mixed with a 4X reducing buffer then boiled for 3 min and then separated by 4–15% Tris-glycine polyacrylamide gel. Colorburst^TM^ markers (Sigma-Aldrich) were used as molecular weight standards. Separated proteins were then transferred onto nitrocellulose membranes in a solution containing 50 mM Tris/HCl, 380 mM glycine and 20% methanol and stained with Ponceau S (ICN Biochemicals, Dublin, OH, United States). Non-specific sites were blocked with 5% dry milk in TTBS and membranes were incubated overnight at 4°C with anti-NF-κB (1:1000; Cell signaling) and anti-IL-1β (1:500; Santa Cruz Tech.) antibodies. Membranes were washed and incubated with a horseradish peroxidase conjugated anti-rabbit IgG secondary antibody (1:2000; Promega, Madison, WI, United States) and then developed using enhanced chemiluminescence reagents (Cat. No. RPN2232, GE Healthcare, Cardif, United Kingdom). Equal protein loading among individual lanes was confirmed by re-probing the membranes with an anti-α-tubulin mouse monoclonal antibody (1:1000; Abcam, Cambridge, MA, United States).

### Statistics

Results are presented as mean ± SEM. Data have been analyzed by using Student’s *t*-test to determine significant differences between groups. Morphological data on OHC survival were evaluated by two-way ANOVA with repeated measures (groups × cochlear turns). *Post hoc* comparisons were assessed with Tukey’s test (Statistica, Statsoft, Tulsa, OK, United States). The level of significance was set at 0.05.

## Results

### Pioglitazone Attenuates Hearing Loss and Promotes OHC Survival

To investigate the protection by pioglitazone on auditory function, we evaluated hearing by ABR in animals prior to noise exposure and 1, 3, 7, 14, and 21 days after single transtympanic administration of 1.2% pioglitazone or matching vehicle. Baseline ABR thresholds did not differ among the experimental groups, consistent with previous data (Fetoni et al., 2015). We included in this study male Wistar rats, in order to better assess the extent of pioglitazone effectiveness, considering that in females sexual and glucocorticoid hormones can positively influence the responses to stress and inflammatory reactions (Canlon et al., 2007; Motohashi et al., 2010; Meltser et al., 2008). However, in a further study, sex differences could be evaluated. Acute noise exposure induced a transient threshold shift of about 40–50 dB in the mid-high frequency region in the first 3 days after insult. Hearing loss partially recovered by 3 weeks, leaving a permanent threshold shift of approximately 25–30 dB. No significant differences were observed between Noise and Noise-vehicle groups (data not shown), indicating that neither the transtympanic injection procedure nor vehicle, *per se*, had a significant effect on auditory function. Moreover, control experiments in normal-hearing animals showed that pioglitazone or vehicle injection, *per se*, did not significantly alter auditory thresholds, monitored up to day 21 after the transtympanic injection (**Supplementary Figure S1**).

**FIGURE 1:**
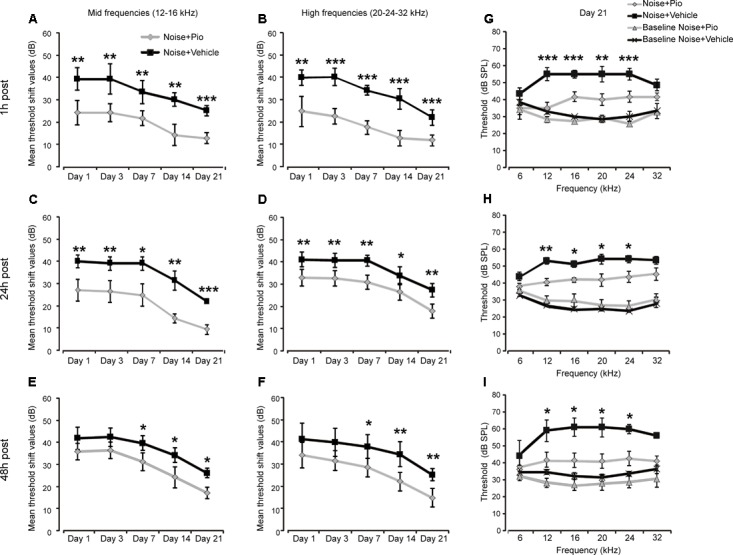
Pioglitazone protects against noise-induced hearing loss. **(A–F)** Graphs show mean threshold shift values (means ± SEM) for mid frequencies (12–16 kHz, **A,C,E**) and high frequencies (20–24–32 kHz, **B,D,F**) measured 1, 3, 7, 14 and 21 days after vehicle (black squares; *n* = 24) or pioglitazone injection (gray diamonds; *n* = 24) in animals exposed to noise and treated 1 h **(A,B)**, 24 h **(C,D)**, or 48 h **(E,F)** after the acoustic trauma. **(G–I)** Graphs show threshold values (means ± SEM) across all frequencies analyzed at day 21 in animals exposed to noise and treated with pioglitazone or vehicle at different time points. Baseline values refer to auditory thresholds estimated prior to pioglitazone or vehicle injection in each condition. Pioglitazone administered 1 h after acoustic trauma provides the major protection, attenuating threshold shift by about 15 dB for mid frequencies and 20 dB for high frequencies. Notably, low frequency (6 kHz) was less affected by acoustic trauma, consistent with our noise exposure protocol (pure tone centered to 10 kHz). Delayed administration (24 and 48 h post noise exposure) shows significant but slightly minor protection (about 10-15 dB). A-F: asterisks indicate significant differences between groups (^∗^*p* < 0.05; ^∗∗^*p* < 0.01; ^∗∗∗^*p* < 0.001). **(G–I)** Asterisks refer to significant differences between Noise + Pio and Noise + Vehicle groups (^∗^*p* < 0.05; ^∗∗^*p* < 0.01; ^∗∗∗^*p* < 0.001).

We explored the efficacy of pioglitazone given immediately (1 h) *vs*. at delayed time points (24 and 48 h) after noise exposure. As indicated in **Figure 1**, animals treated 1 h after noise exposure showed a significantly reduced threshold shift of about 20–30 dB at mid-high frequencies in pioglitazone-treated ears, compared to about 40–50 dB threshold shift observed in the contralateral vehicle-treated ears (**Figures 1A,B**). The reduction in threshold shift was evident at day 1 following pioglitazone treatment and persisted at all time points throughout the 3 week follow up period (**Figures 1A,B,G**). Notably, by day 21 post-treatment, pioglitazone-treated ears had recovered to within 10 dB of baseline at mid and high frequencies, while vehicle treated ears exhibited a residual hearing loss of approximately 20–30 dB (**Figure 1G**).

Previous studies have shown that early drug intervention prior to or shortly after noise, is critically required in order to successfully target early pathological mechanisms. Therefore, it was of interest to assess delayed treatment. Pioglitazone, when administered 24 h after noise exposure was able to reduce the threshold shift of treated *vs*. control ears by approximately 10–15 dB better at mid frequencies than high frequencies (**Figures 1C,D**) which persisted through the follow up period. As shown in **Figure 1H**, at the end of treatment, a protective effects of about 15 dB persisted. Pioglitazone treatment at 48 h attenuated threshold shift by about 10–15 dB that was evident up to the last time point at 3 weeks after noise exposure (**Figures 1E,F,I**).

**FIGURE 2:**
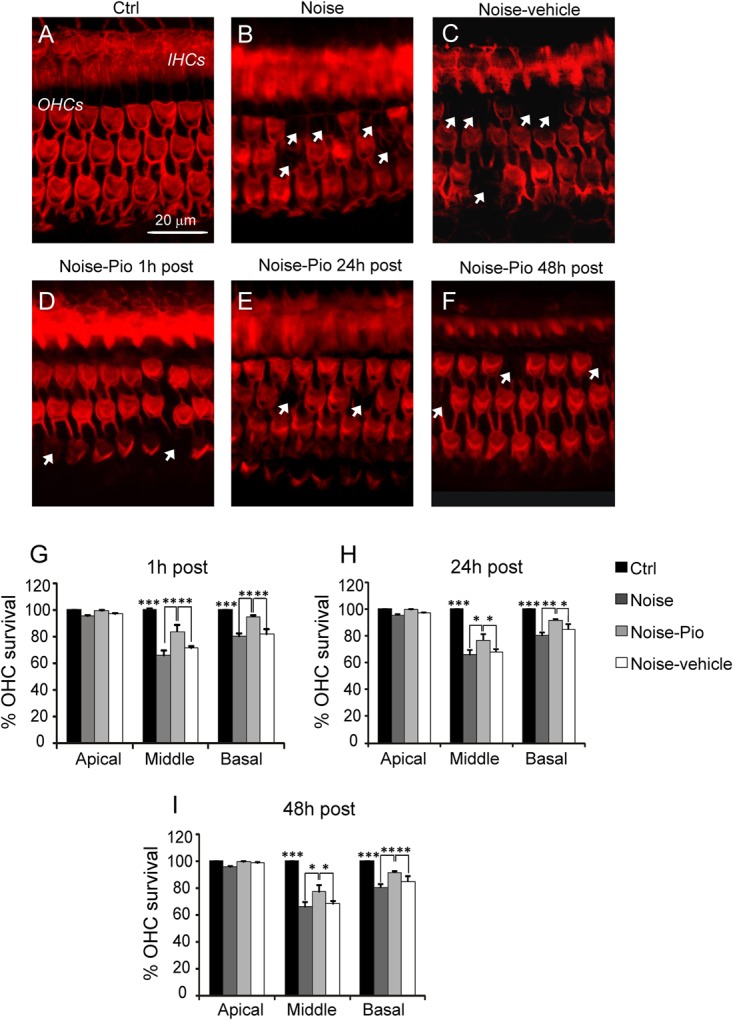
Morphological evaluation and hair cell count. **(A–F)** Representative images of surface preparation of the organ of Corti showing F-actin distribution in the middle-basal cochlear turn. A typical distribution of three rows of outer hair cells (OHCs) and one row of inner hair cells (IHCs) is shown in **(A)**. Noise exposure **(B)** caused OHC loss (dark spots, indicated with arrows). Pioglitazone significantly reduced OHC death **(D–F)** with respect to Noise-vehicle condition **(C)**, specifically if administered 1 h after noise insult **(D)**. Scale bar: 20 μm. **(G–I)** Cochleograms (means ± SEM) showing percentage of OHC survival in the three different administration schedules. Noise induced about 40% and 20% of cell death in the middle and basal turns respectively. Pioglitazone administration attenuates cell death of about 20–25%. Asterisks indicate significant differences between groups (^∗^*p* < 0.05; ^∗∗^*p* < 0.01; ^∗∗∗^*p* < 0.001; *n* = 6 animals/group).

Furthermore, we examined OHC viability in surface preparations of the basilar membrane of organ of Corti prepared at 3 weeks following noise exposure and treatment. **Figure 2** illustrates Rh-Ph staining and OHC counts in Ctrl, Noise, Noise-vehicle and Noise-Pio groups for the 1, 24 and 48 h treatment interventions after acoustic trauma. Noise exposure induced mostly loss of OHCs, which was reflected by dark spots, phalangeal scars and disappearance of both cuticular plates and hair bundles (arrows) limited mainly to the middle-basal cochlear turns (**Figure 2B**). Noise exposure alone decreased OHC count in the middle turn to approximately 60% of remaining cells as compared to the Ctrl group (**Figures 2B,G–I**). The Noise-vehicle group showed similar hair cell loss, with no significant differences with respect to Noise group (**Figures 2C,G–I**). Pioglitazone, administered 1 h after acoustic injury, significantly reduced the OHC loss in the middle turn, where cell count was approximately 85–90% *vs*. the Noise group (**Figures 2D,G**). Pioglitazone, administered 24 or 48 h after acoustic trauma, promoted OHC survival reaching approximately 75 and 85% *vs*. controls in the middle-basal turn (**Figures 2E,F,H,I**), which was slightly less effective as compared to the 1 h post treatment (**Figure 2G**). No significant differences were observed among groups in the apical turn (**Figures 2G–I**), accordingly to the electrophysiological data. The effects of pioglitazone promote OHC survival support the functional improvement in hearing capacity. The registration of distortion product otoacoustic emission (DPOAEs), generated by active OHCs can provide a further evidence of the effectiveness of pioglitazone on the sensory epithelia, main target of acoustic trauma (Fetoni et al., 2016). Together, these results show that early pioglitazone intervention, when it may counteract the early oxidative stress response, is most effective to prevent NIHL. However, the observation that delayed treatment was also effective, although to a lesser extent, suggests that pioglitazone may target additional mechanisms.

### Pioglitazone Reduces Cochlear Oxidative Stress Induced by Noise Exposure

To assess the protective effects of pioglitazone against noise-induced oxidative stress, we performed immunofluorescence analyses on cochlear sections to detect superoxide anion radicals (DHE staining) and the lipid peroxidation product 8-Isoprostane.

**FIGURE 3:**
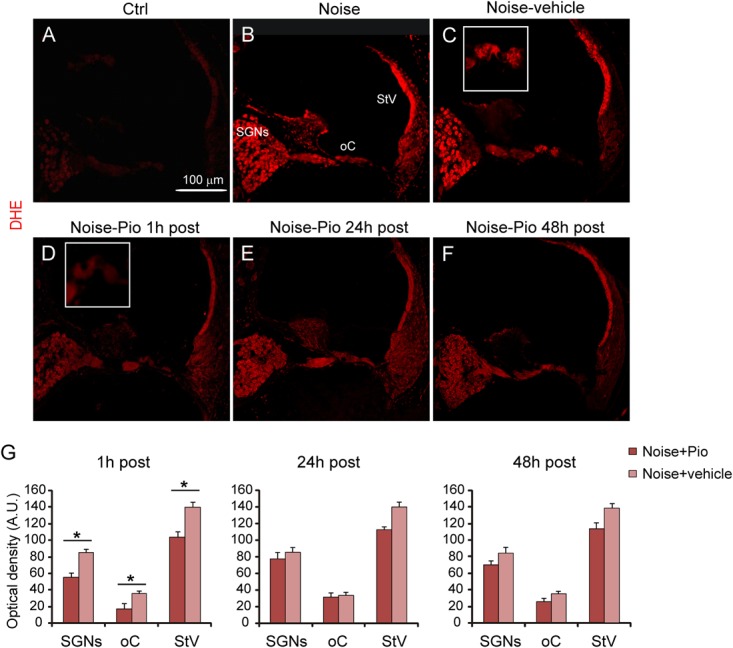
Early pioglitazone administration reduces superoxide amount in the cochlea. **(A–F)** Representative images of cochlear cryo-sections (middle-basal turns) stained with DHE (red fluorescence). Insets of the organ of Corti are shown in **(C,D)**. Superoxide fluorescence was faint in the cytoplasm of control cochleae **(A)**. Noise exposure increased superoxide amount, mainly in spiral ganglion neurons, organ of Corti and *stria vascularis* **(B)**. No difference in superoxide amount was found between Noise and Noise-vehicle conditions **(B,C)**. When administered 1 h after noise exposure, pioglitazone significantly decreased superoxide expression in the main cochlear structures **(D)**. Administration of either 24 h **(E)** and 48 h **(F)** post-noise also reduced superoxide but to a lesser extent as compared to earlier administration. Scale bar: 100 μm. **(G)** Histograms (means ± SEM), related to Noise-Pio and Noise + vehicle groups, show quantification of fluorescence intensity (A.U., arbitrary units) in the principal cochlear structures: spiral ganglion neurons (SGNs), organ of Corti (oC) and *stria vascularis* (StV). Asterisks indicate significant differences between groups (^∗^*p* < 0.05; *n* = 12 slices selected randomly from 6 animals for each experimental group).

**Figure 3** illustrates the level of superoxide in cochlear cryosections detected by DHE fluorescence. Red fluorescence was faint in control cochleae from animals not exposed to noise (**Figure 3A**). Noise exposure in both control animals (**Figure 3B**), as well as in Noise-vehicle treated animals (**Figure 3C**), dramatically increased cochlear superoxide levels, as reflected by increased DHE staining, detected particularly in SGNs, the organ of Corti and the *stria vascularis*, as also confirmed by fluorescence intensity quantification (**Figure 3G**). No significant differences were observed among Noise-vehicle 1, 24 and 48 h post groups (data not shown). The increased oxidative stress induced by noise exposure was significantly blocked by pioglitazone in ears treated 1 h after acoustic trauma in all cochlear structures (**Figures 3D,G**). Delayed administration of pioglitazone to 24 or 48 h after noise only slightly reduced DHE signal (**Figures 3E,F**). According to the optical density analyses, significant differences in superoxide expression were observed in all cochlear structures only in the samples from animals treated shortly (1 h) after noise exposure. Delayed treatments did not lead to significant differences in all structures even if DHE expression was slightly more evident in the *stria vascularis* (**Figure 3G**).

**FIGURE 4:**
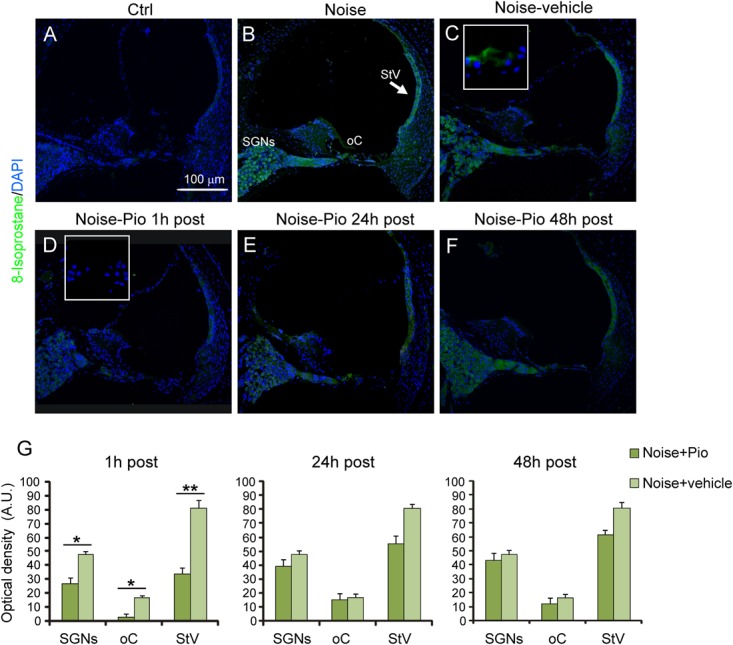
Pioglitazone reduces lipid peroxidation when administered 1 h after noise exposure. **(A–F)** Representative images of cochlear cryo-sections (middle-basal turns) stained with a marker of lipid peroxidation (8-Isoprostane, green fluorescence) and stained with DAPI (blue fluorescence). Insets of high magnification of the organ of Corti are shown in **(C,D)**. Noise exposure induces an increase of lipid peroxidation in all cochlear structures **(B)**, with no difference with respect to animals exposed to noise and treated with vehicle **(C)**. Lipid peroxidation was absent in the cytoplasm of control cochleae **(A)**. Noise exposure increased 8-Isoprostane amount, mainly in spiral ganglion neurons, organ of Corti and specifically *stria vascularis* (see arrow in **B**). No difference was found between Noise and Noise-vehicle conditions **(C)**. When administered 1 h after noise exposure, pioglitazone significantly decreased 8-Isoprostane expression **(D)**. Administration 24 h **(E)** and 48 h **(F)** post noise showed small but detectable effect in counteracting lipid peroxidation. Scale bar: 100 μm. **(G)** Histograms (means ± SEM) show quantification of fluorescence intensity (A.U., arbitrary units) as described in **Figure 3**. SGNs, spiral ganglion neurons; oC, organ of Corti; StV, *stria vascularis*. Asterisks indicate significant differences between groups (^∗^*p* < 0.05; ^∗∗^*p* < 0.01; *n* = 12 slices selected randomly from 6 animals for each experimental group).

To determine the level of lipid peroxidation, we performed 8-Isoprostane immunofluorescence in cochlear sections. **Figure 4** illustrates that, similarly to the results for DHE, noise exposure induced the production of 8-Isoprostane, indicating a significant lipid peroxidative damage as a consequence of noise trauma (**Figure 4B** *vs*. **Figure 4A**). The increase in 8-Isoprostane was most evident in SGNs, the organ of Corti and the *stria vascularis*, the same structures which had also exhibited the greatest increase in superoxide. As reported for DHE staining, no significant differences were observed among Noise-vehicle 1, 24 and 48 h post groups (data not shown). Notably, pioglitazone administered 1 h after noise exposure, completely blocked the increase in 8-Isoprostane fluorescence *vs*. vehicle-treated ears (**Figures 4C** *vs*. **Figure 4D**), in particular in the *stria vascularis*. Delayed treatment with pioglitazone (24 or 48 h) also reduced 8-Isoprostane, with residual signal present mainly in the SGNs (**Figures 4E,F**). Fluorescence signal quantification in the SGNs, organ of Corti and *stria vascularis* confirmed that the efficacy of pioglitazone to counteract lipid peroxidation induced by noise was evident primarily when administered 1 h after trauma (**Figure 4G**).

### Pioglitazone Reduces the Noise-Induced Inflammatory Response

**FIGURE 5:**
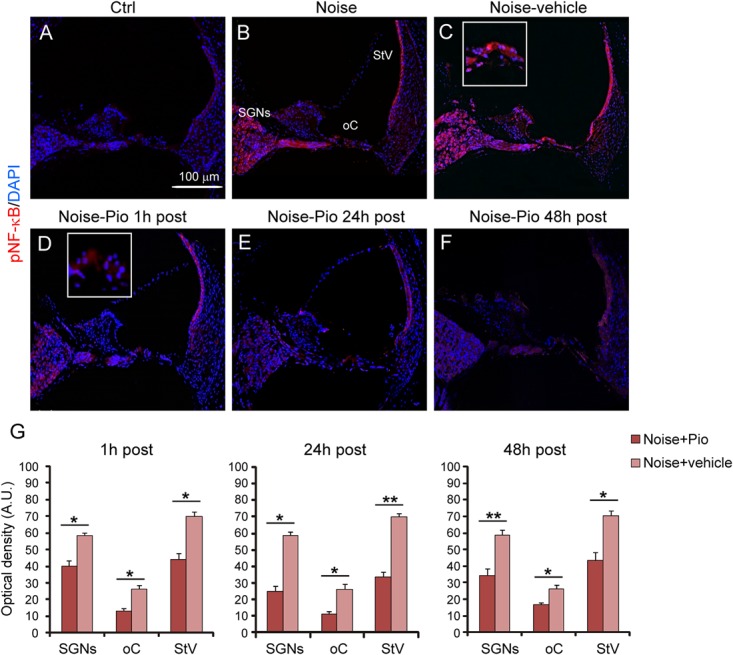
Pioglitazone reduces cochlear expression of NF-κB. **(A–F)** pNF-κB (red fluorescence) and DAPI staining (blue fluorescence) in cochlear cryo-sections (middle-basal turns) of all experimental groups. Insets of high magnification of the organ of Corti are shown in **(C,D)**. NF-κB expression increased after noise exposure in all cochlear structures **(B)** with no differences between Noise and Noise-vehicle groups **(C)**. Local delivery of pioglitazone attenuated NF-κB phosphorylation when administered 1 h post noise **(D)** as well as when administered either 24 and 48 h post noise **(E,F)**. **(G)** Histograms (means ± SEM) show quantification of fluorescence intensity (A.U., arbitrary units) as described in **Figure 3**. SGNs, spiral ganglion neurons; oC, organ of Corti; StV, *stria vascularis*. Asterisks indicate significant differences between groups (^∗^*p* < 0.05; ^∗∗^*p* < 0.01; *n* = 12 slices selected randomly from 6 animals for each experimental group).

**FIGURE 6:**
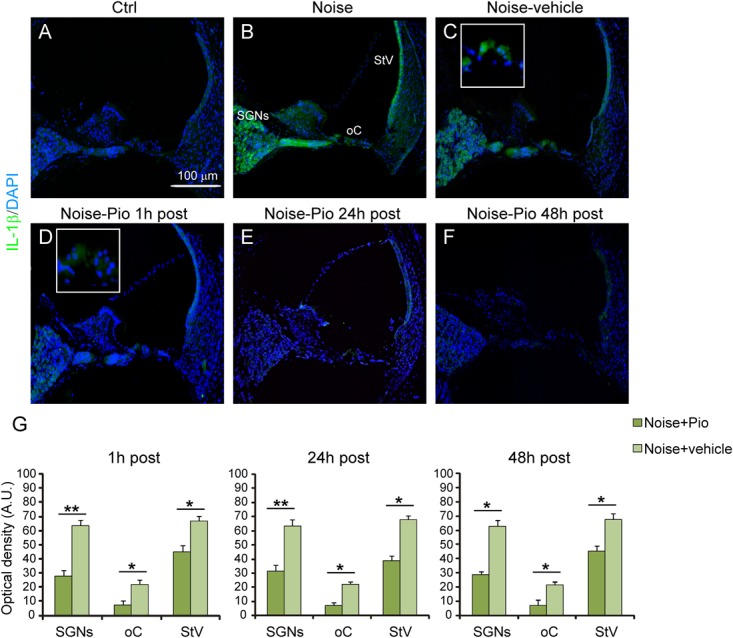
Pioglitazone counteracts the increase of IL-1β expression. **(A–F)** Representative images of IL-1β expression (green fluorescence) in cochlear cryo-sections (middle-basal turns) stained with DAPI (blue fluorescence). Insets of high magnification of the organ of Corti are shown **(C,D)**. The IL-1β expression increase observed after noise exposure **(B)** was significantly reduced after pioglitazone treatment **(D–F)**. No difference was found between Noise and Noise-vehicle groups **(C)**. Scale bar: 100 μm. **(G)** Histograms (means ± SEM) show quantification of fluorescence intensity (A.U., arbitrary units) as described in **Figure 3**. SGNs, spiral ganglion neurons; oC, organ of Corti; StV, *stria vascularis*. Asterisks indicate significant differences between groups (^∗^*p* < 0.05; ^∗∗^*p* < 0.01; *n* = 12 slices selected randomly from 6 animals for each experimental group).

**FIGURE 7:**
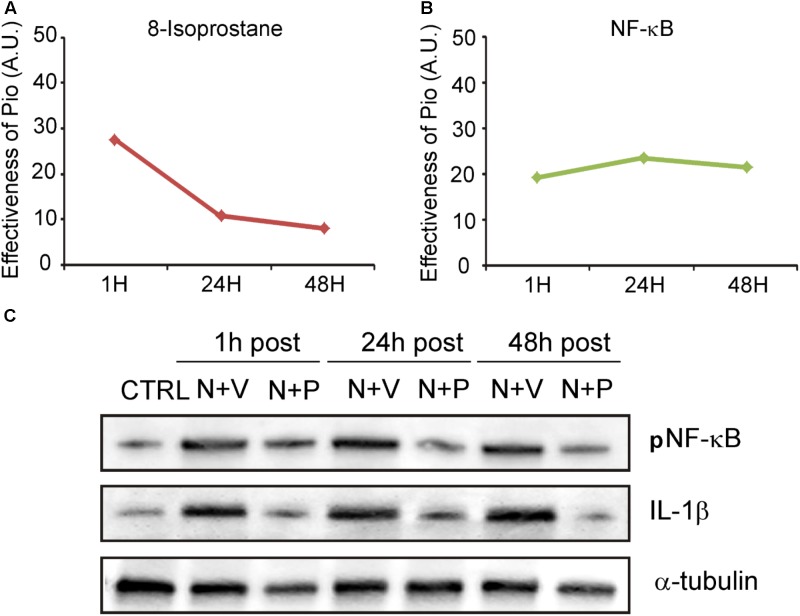
Pioglitazone targets the oxidative/inflammatory interplay in cochlear damage. **(A,B)** Graphs show the shift/decrease of fluorescence intensity (measured by comparing Noise-vehicle *vs*. Noise-Pio conditions) for the different immunofluorescence analysis (8-Isoprostane and NF-κB), at the different drug administration schedules (1, 24 or 48 h post noise exposure). Data are expressed as means ± SEM (A.U., arbitrary units). **(C)** Representative immuno-reactive bands for pNF-κB and IL-1β in controls and animals exposed to noise and treated with pioglitazone (N + P; *n* = 6) or vehicle (N + V; *n* = 6) at the different time points (1, 24, or 48 h after noise exposure) 7 days after administration. Reduction of both NF-κB and IL1-β was clearly detected 7 days after drug treatment.

We then evaluated the effects of pioglitazone on NF-κB, a critical regulator of the inducible expression of genes in inflammation and on IL-1β, a pro-inflammatory cytokine implicated in cochlear damage. **Figures 5, 6** show pNF-κB and IL-1β immunofluorescence in cochlear sections from control and noise-exposed animals, as well as noise-exposed animals treated with vehicle or pioglitazone 1, 24 or 48 h after acoustic trauma. No significant differences were observed among Noise-vehicle 1, 24 and 48 h post groups (data not shown). Noise exposure dramatically induced NF-κB phosphorylation (red fluorescence) in all cochlear structures in control and vehicle treated ears (**Figures 5B,C**). Pioglitazone effectively blocked the noise-induced increase in pNF-κB at all treatment intervals following acoustic trauma (**Figures 5D–F**), as also confirmed by optical density analysis in the principal cochlear structures (**Figure 5G**). Similar results were observed for IL-1β. Noise exposure significantly upregulated IL-1β, with the most intense signal detected in the SGNs and the *stria vascularis* (**Figures 6B,C**). Pioglitazone effectively blocked the noise-induced increase in IL-1β, with no signal detected at any time point in the SGNs or in the organ of Corti and only faint signal detected in the *stria vascularis* at day 7 post-treatment (**Figures 6D–G**). Interestingly, these results suggest that, while delayed treatment with pioglitazone is less effective *vs*. early treatment to reduce oxidative stress (**Figure 7A**), even late administration of pioglitazone is able to block the noise-induced inflammatory response, as reflected by attenuation of pNF-κB (**Figure 7B**).

To extend and confirm the immunofluorescence results, we performed western blot analysis in cochlear lysates. **Figure 7C** shows pNF-κB and IL-1β immunoreactive bands in protein samples from cochleae isolated from normal hearing controls and noise-exposed animals treated with pioglitazone or vehicle. Data are shown for the different drug administration schedules (1, 24 or 48 h after noise exposure) at day 7 after drug treatment. Similar to the immunofluorescence results, pioglitazone inhibited the noise-induced increase of both NF-κB phosphorylation and IL-1β (**Figure 7C**). The western blot data, consistent with the immunofluorescence results, confirmed that pioglitazone effectively reduces inflammatory pathways following both early or delayed administration after noise trauma.

## Discussion

To date, no drugs have been specifically developed and approved for the treatment of hearing loss-related disorders, despite extensive research on preventative approaches. The results presented here in a NIHL *in vivo* model, demonstrate that, pioglitazone, the PPARγ agonist, may represent an effective and innovative therapeutic strategy for hearing loss-related diseases such as sensorineural hearing loss, aging and ototoxicity. Our results provide evidence that pioglitazone reduces noise-induced inflammatory responses and cochlear oxidative stress. Specifically, a single transtympanic injection of pioglitazone at different time points after noise exposure decreased: (i) ABR threshold shift at all time points analyzed; (ii) cell death in the organ of Corti; (iii) cochlear superoxide production and lipid peroxidation and (iv) cochlear NF-κB and IL-1β inflammatory response.

### Transtympanic Pioglitazone Attenuates NIHL

Many of the immediate responses in the cochlea to acute trauma (ROS generation, lipid peroxidation, HC apoptosis) are rapid and transient (Henderson et al., 2006). Therefore, formulations and methods to provide drugs as quickly as possible to the site of injury are desired. There have been efforts to achieve this through devices and sustained-release drug delivery systems such as the use of nanoparticles or biodegradable hydrogels, that can increase the residence time of a given drug in the middle ear and provide rapid controlled drug delivery to the inner ear. A promising approach is represented by the thermosensitive gels. These polymers provide the advantage of a transition to gel in the tympanic cavity at body temperature increasing the residence time of the drug in the middle ear (Liu et al., 2014). Our results show that the pioglitazone gel formulation was safe, given that no threshold elevation or cochlear cytotoxic effects were found in control animals that underwent pioglitazone or vehicle injection (**Supplementary Figure S1**). At functional and morphological level, the 1.2% pioglitazone gel suspension was able to effectively protect hearing at mid-high frequencies and to limit loss of OHCs at the basal/middle turn damaged by noise exposure after a single drug injection. The best pioglitazone protection against NIHL was achieved when drug administration occurred 1 h after acoustic trauma, consistent with interference in early mechanisms of cochlear response to trauma (**Figure 1**). Interestingly, even delayed administration of pioglitazone 24 or 48 h after noise exposure showed a reduced but significant effect to protect hearing from noise trauma (reduced threshold shift elevation of about 10–15 dB, **Figures 1A,B**). Therefore, in a clinical perspective, this molecule is effective in the most common condition in which patients require treatment after noise insult. Preventive effects of pioglitazone on subjects exposed to risk factors for NIHL could also be hypothesized, however, further experimental studies should be undertaken.

### Inflammation and Oxidative Stress Are Attenuated by Pioglitazone

In our model, the local delivery of pioglitazone had potent anti-inflammatory effects in the cochlea through reduced NF-κB and IL-1β cochlear expression (**Figures 5, 6**). This anti-inflammatory effect persisted at all administration time schedules and was potentially responsible for the significant protection against NIHL in animals with delayed treatment with pioglitazone at 24 and 48 h after noise injury. Indeed, several inflammation-related genes and proteins have been implicated in the cochlear response both to acute noise exposure (Yamamoto et al., 2009; Gratton et al., 2011; Nakamoto et al., 2012; Fujioka et al., 2014) and following chronic environmental noise exposure (Tan et al., 2016). Indeed, in our model of acute noise exposure, the expression of inflammatory regulators, IL-1β and phospho-NF-κB, increased. NF-κB is a major transcription factor sequestered in the cytoplasm with direct interaction with the inhibitory kappa B (IkB) family (Hayden and Ghosh, 2004, 2008; Gloire et al., 2006). Under stress conditions, the IkB is phosphorylated, releasing NF-κB dimer which translocates into the nucleus and drives gene expression (Hayden and Ghosh, 2011, 2012, 2014; Luan et al., 2013). NF-κB governs the transcription of a wide array of genes that are of central importance in inflammation and immunity (Hayden and Ghosh, 2008; Vallabhapurapu and Karin, 2009). In this study, pioglitazone treated animals exhibited a decrease of NF-κB phosphorylation and suppression of IL-1β in all cochlear structures. In support of a likely role in cochlear protection, it has been suggested that NF-κB signaling plays a role in the effects of pioglitazone to protect against focal cerebral ischemia in rats (Zhang et al., 2011) and in several rodent models of neurological diseases (Nakamura et al., 2007; Park et al., 2007; Swanson et al., 2011). In these diverse models, pioglitazone demonstrated neuroprotective and/or restorative effects, mainly associated with modulation of inflammation. Notably, pioglitazone upregulated the expression of IκB in the brain in an experimental model of autoimmune encephalomyelitis (Feinstein et al., 2002) and in cultured neurons (Zhang et al., 2011).

In addition to the beneficial effects on inflammation, pioglitazone effectively reduced oxidative stress, reflected by ROS and lipid peroxidation decrease. Indeed, the oxidative stress induced by noise exposure (**Figures 3, 4**, superoxide and 8-Isoprostane production) was counteracted by pioglitazone both when administered 1 h after the acoustic trauma and, to a lesser extent, when given 24 or 48 h after noise exposure. Our *in vivo* data are consistent with recent data obtained *in vitro* in a gentamicin ototoxicity model where it reported that pioglitazone provided protection against ROS increase, lipid peroxidative damage and HC apoptosis by upregulating redox pathway genes and by potentiating the cell own antioxidant defenses (Sekulic-Jablanovic et al., 2017). In the cited report, pioglitazone rescued hair cells from gentamicin oxidative toxicity and apoptosis by targeting PPARγ and PPARα proteins expressed in cochlear structures. Our data presented here imply that these mechanisms may be also relevant *in vivo* and offer the potential to achieve clinically meaningful effects for pioglitazone in the treatment of hearing loss. Although further experimentation is needed to explore PPARγ activation by pioglitazone in the cochlea, our data could also be consistent with previous reports in which pioglitazone effectively enhanced enzymatic antioxidant defenses in the brain (Shimazu et al., 2005; Suge et al., 2012) and with the demonstration that PPARγ is able to protect cells from apoptosis through strong anti-inflammatory and antioxidant effects (Abdallah, 2010; Barroso et al., 2013). Notably, at molecular level, PPARγ activation stabilizes mitochondria, averts oxidative damage and supports neuronal survival (Fuenzalida et al., 2007). Moreover, it has been reported that pioglitazone specifically binds to an additional target within mitochondrial membranes, a class of proteins (mitoNEET), required for increasing mitochondrial inner membrane integrity and for ROS homeostasis (Paddock et al., 2007; Mittler et al., 2018). Mitochondria are the major source of ROS generated in cochlear cells after injury and, thus, the robust protective effects of pioglitazone are not surprising and consistent with a wide body of literature (Henderson et al., 2006; Lenaz et al., 2007; Fetoni et al., 2015). In order to explore the antioxidant effects in the cochlea in the NIHL model, the timing of treatment was crucial. In our model, the ability of pioglitazone to reduce oxidative imbalance was most effective in the early time window of administration (1 h after noise exposure), which is consistent with the known kinetics of cochlear ROS generation in the cochlea (Seidman et al., 1999; Ohinata et al., 2000; Jaumann et al., 2012). Our previous reports have shown that ROS are detected early, within the first 2 h following noise trauma and remain elevated up to 7.5 h. Lipid and protein peroxidation occurs around ∼4 h following noise insult (Maulucci et al., 2014). Thus, antioxidant rescue by pioglitazone was greatest when administered in an early time window, which prevented the initial formation of ROS which, when triggered, can promote ongoing cellular damage. These data are consistent with similar results that have been obtained in NIHL animal models employing polyphenols as treatments (Fetoni et al., 2015, 2014a).

Altogether, the protective effects of pioglitazone appear to be related to anti-inflammatory and anti-oxidative mechanisms, suggesting a possible ROS/NF-κB signaling crosstalk/relationship in the cochlea. It has been reported that ROS interact with NF-κB signaling pathways in many ways. The transcription of NF-κB dependent genes has been shown to influence the levels of ROS in the cell and, in turn, the levels of NF-κB activity are also influenced by the levels of ROS (Gloire et al., 2006; Morgan and Liu, 2011; Blaser et al., 2016). Interestingly, in our study the antioxidant effects of pioglitazone were evident mostly during the peri-traumatic period (1 h post-noise administration) when ROS production begins to rise, while the anti-inflammatory effect persisted for a longer time after noise exposure (24 and 48 h administration), when ROS was already declining (Kurabi et al., 2017). Thus, it is plausible that, soon after noise exposure, pioglitazone may negatively regulate ROS/NF-κB crosstalk (see **Figures 7A,B** illustrating decrease of both lipid peroxidation and inflammation). Later on (24 and 48 h after noise exposure), when ROS/NF-κB crosstalk is maximal and ROS production has declined, pioglitazone exhibits the majority of its anti-inflammatory effects (see **Figure 7**). NF-κB redox regulation has been intensely studied in several cell-types and biological conditions and, although it is now clear that NF-κB activation mechanism relies mainly on IkB activation, the redox-sensitive pathways triggering this activation, as well as the ROS/NF-κB interplay are reported to be quite different depending on the cell-type (Morgan and Liu, 2011).

Furthermore, a remarkable effect of pioglitazone was revealed in the cochlear vascular structure, the *stria vascularis*, where it reduced the 8-Isoprotane fluorescence signal, in particular in the animals treated 1 h after noise exposure. Given that isoprostanes can potentially lead to reduced cochlear blood flow (Seidman et al., 1999; Ohinata et al., 2000; Jaumann et al., 2012), these data suggest that pioglitazone can potentially activate the hypoxia response pathway associated with cochlear redox unbalance induced by noise exposure (Zhang et al., 2014). Further work will be needed to explore this hypothesis.

In summary, these results reveal that pioglitazone has multiple protective mechanisms in the cochlea, favorably affecting oxidative stress and inflammation. However, even if pioglitazione acts as an agonist of PPARγ in several conditions, further studies are needed to demonstrate their activation in the protection against cochlear stress status. Early intervention is expected to provide the greatest protection against exogenous insults, while later favorable effects on inflammation may contribute to either greater or sustained efficacy and will also extend the window of opportunity for effective therapy. These factors are important in the treatment of hearing loss after acute noise trauma or sudden sensorineural hearing loss, where rapid intervention is critical. Pioglitazone, via its multiple favorable mechanisms of cochlear protection, may offer an attractive option for the treatment of sensorineural hearing loss.

## Author Contributions

FP: data collection and interpretation. AF: study design, data collection, data analysis direction and supervision, and wrote the manuscript. RR: data collection. MW: study design. CG: data quality control. DT: data analysis direction and supervision, and wrote the manuscript. GP: study design, data quality control, and manuscript editing.

## Conflict of Interest Statement

MW is an employee of Strekin AG and receives salary and stock options. The funders had no role in data collection and interpretation, or the decision to submit the work for publication. This does not alter our adherence to Frontiers in Pharmacology policies on sharing data and materials. The remaining authors declare that the research was conducted in the absence of any commercial or financial relationships that could be construed as a potential conflict of interest. The reviewer AB and handling Editor declared their shared affiliation.
