# Supplementary figures and images for: Pioglitazone Represents an Effective Therapeutic Target in Preventing Oxidative/Inflammatory Cochlear Damage Induced by Noise Exposure
Source: Front Pharmacol. 2018 Oct 8;9:1103. doi: 10.3389/fphar.2018.01103 (PMC6187064; doi:10.3389/fphar.2018.01103)

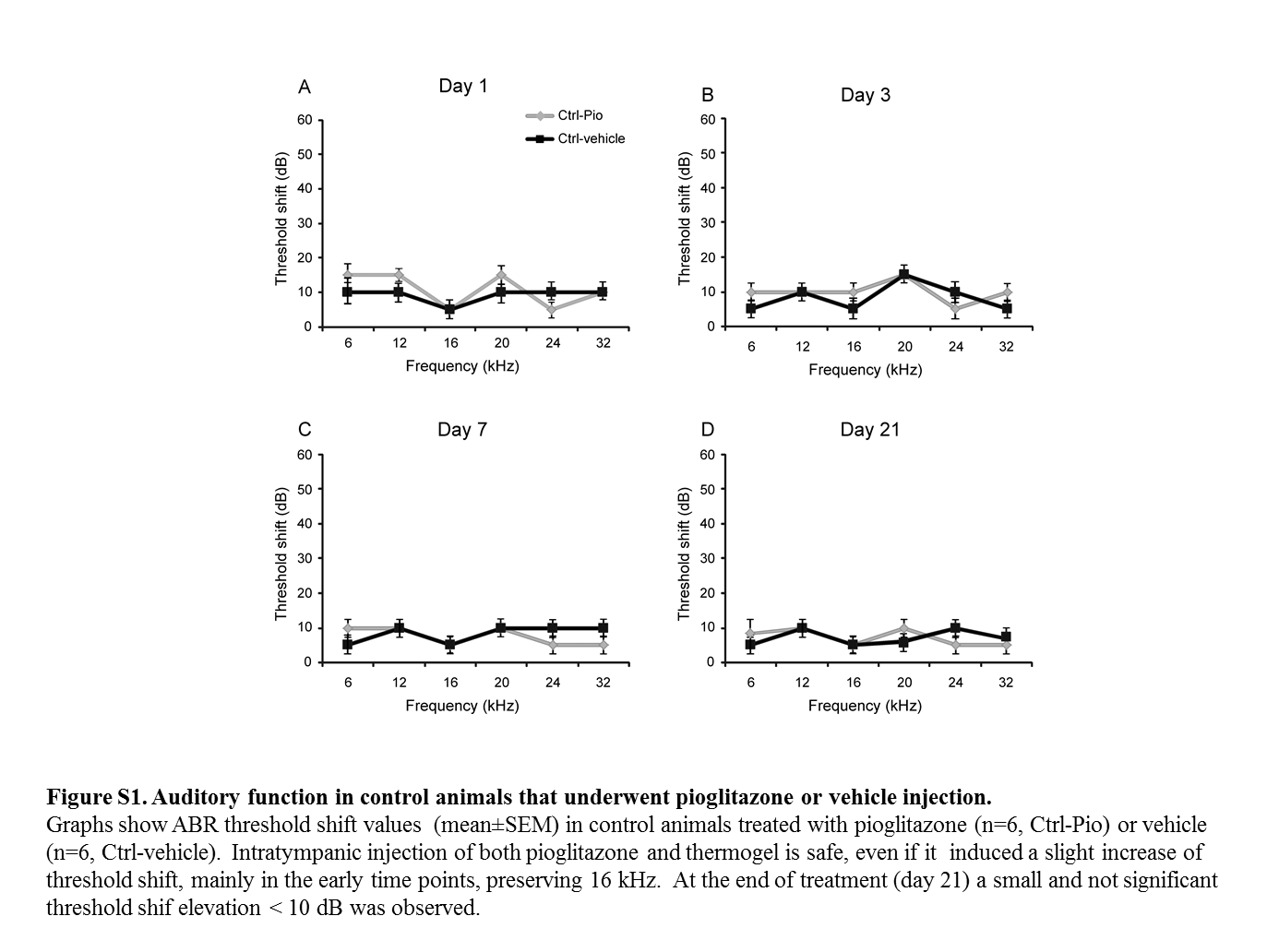

Supplement: Supplementary file 1 [file Image_1.TIF]
